# Preparation and characterization of fast-curing powder epoxy adhesive at middle temperature

**DOI:** 10.1098/rsos.180566

**Published:** 2018-08-15

**Authors:** Jie Xu, Jiayao Yang, Xiaohuan Liu, Hengxu Wang, Jingjie Zhang, Shenyuan Fu

**Affiliations:** 1 Zhejiang Provincial Collaborative Innovation Center for Bamboo Resources and High-efficiency Utilization, 666 Wusu Street, Hangzhou 311300, People's Republic of China; 2 School of Engineering, and Key Laboratory of Wood Science and Technology of Zhejiang Province, Zhejiang A&F University, 666 Wusu Street, Hangzhou 311300, People's Republic of China

**Keywords:** epoxy resin, powder epoxy adhesive, 2-methyl imidazole, curing reaction kinetic

## Abstract

At present, the disadvantage of powder epoxy adhesive is the limited application area. In order to widen the application range of powder epoxy adhesive from heat-resistant substrates (such as metals) to heat-sensitive substrates (such as plastic products, cardboard and wood), it is necessary to decrease the curing temperature. In this article, a series of fast-curing powder epoxy adhesives were prepared by the melt blending method with bisphenol A epoxy resin (E-20), hexamethylenetetramine (HMTA) as a curing agent and 2-methylimidazole (2-MI) as an accelerant. The structure and properties of the E-20/HMTA/2-MI systems were characterized by Fourier transform infrared, thermogravimetric analysis, dynamic mechanical analyser and differential scanning calorimetry (DSC). 2-MI added into the E-20/HMTA systems can simultaneously enhance toughness, tensile strength, glass transition temperature (*T*_g_) and thermal stability in comparison with the E-20/HMTA systems. The best mechanical properties were obtained at 100/8/0.6 weight ratio of the E-20/HMTA/2-MI systems. DSC experiments revealed that the exothermic peak of the E-20/HMTA/2-MI system was about 55°C lower than that of the E-20/HMTA system. The activation energy of the cure reaction was determined by both Kissinger's and Ozawa's methods at any heating rates. The activation energy and pre-exponential factor were about 100.3 kJ mol^−1^ and 3.57 × 10^11^ s^−1^, respectively. According to the KAS method, the curing time of the E-20/HMTA/2-MI systems was predicted by evaluating the relationship between temperature and curing time.

## 1. Introduction

Bisphenol A epoxy resins (DGEBAs) are a unique class of thermoset polymers, which have been gaining ground since their commercial debut in the 1940s [1]. Epoxy resins have found a myriad of applications as in coatings, adhesives, construction and electrical engineering for decades [2], owing to their superior mechanical and chemical properties, such as high tensile, good chemical and solvent resistance, and high heat distortion temperatures [3–5].

Powder epoxy resins, a novel kind of epoxy resins without any solvent, have recently received considerable attention because of the imposition of increased legislative restrictions on the emission of organic solvents to the atmosphere [6]. Additionally, powder epoxy resins have many advantages, such as non-toxicity, ease of use and high recycling rate. In practice, epoxy adhesives that cure fast at mild temperatures are needed because excessive heat cannot be applied to the peripheral components and adherend [7]. On the other hand, the demand for increased manufacturing throughput is driving cycle times down from hours to several minutes [8]. In consideration of energy saving and consumption reduction, a fast-curing powder epoxy system deserves to be explored.

Generally, resin curing time can be lowered by introducing and/or improving the catalytic behaviour of epoxy cross-linking with the curing agent or accelerator [9]. Many different kinds of curing agents or accelerators are applied to epoxy resins, and they are mainly divided into phenol formaldehyde resins, acid anhydrides, amines and imidazoles [3,10]. Among them, imidazoles have recently been paid attention because they can initiate the homopolymerization of the epoxy compounds [3,11,12]. Both nucleophilic and base-catalysed processes can be considered, and these properties can be altered by substituted groups on the imidazole ring [13]. When imidazoles are added into the epoxy resin for 1 h to a few days, the epoxy resin will transform into solid at low temperature, suggesting comparatively high reactivity of imidazoles [14].

Imidazole-based curing agents have been attracting increasing research interests. Pin *et al.* [15] earlier reported MHHPA cured epoxidized linseed oil catalysed by 2-methylimidazole (2-MI) with high thermal stability. The addition of 2-MI remarkably reduced the curing temperature of the reaction, and increased the mechanical, thermal properties of the blends because these were more active initiators for fast cross-linking [16]. Besides, 2-MI was more effective than 1-methylimidazole, 2-ethylimidazole, 2-phenylimidazole, 2-ethyl-4(5)-methylimidazole or 1-(2-cyanoethyl)-2-ethyl-4(5)-methylimidazole in initiating the polymerization of epoxy resin [3,17]. To the best of our knowledge, the incorporation of 2-MI into powder epoxy resin for developing fast-curing powder epoxy adhesive is rare.

The key aim of this paper is thus to deepen our understanding of the fast-curing powder epoxy adhesive [18]. Bisphenol A epoxy resin (E-20), hexamethylenetetramine (HMTA) and 2-MI are chosen to prepare fast-curing powder epoxy adhesive, and study its mechanical properties, structural characteristics, thermal stability and curing kinetics. The results of this study can be used to make recommendations for the optimal curing parameter of the E-20/HMTA/2-MI system.

## 2. Material and methods

### 2.1. Materials

Hexamethylenetetramine (assay greater than 99.5%, molecular formula: C_6_H_12_N_4_, molecular weight (g): 140.18) and 2-methyl imidazole (molecular formula: C_4_H_6_N_2_, molecular weight (g): 82.10) were supplied by Sinopharm Chemical Reagent Co., Ltd (Shanghai, China). Epoxy resin (E-20) was supplied by Huangshan Jinfeng Industrial Co., Ltd.

### 2.2. Preparation of samples

**Figure 1.**
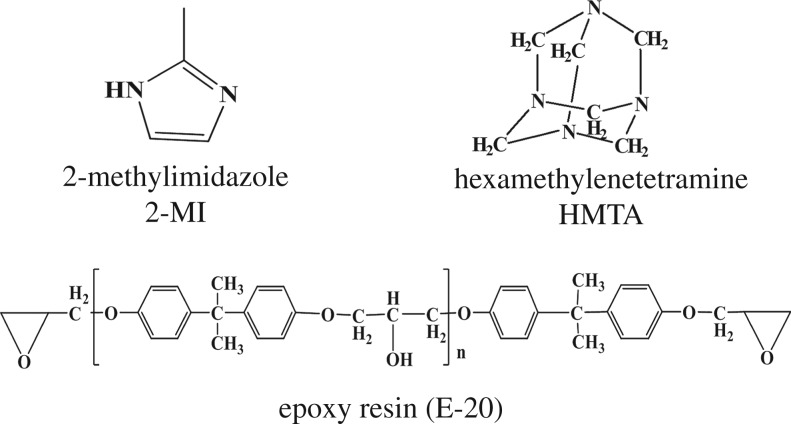
Chemical structures of the E-20, HMTA and 2-MI.

**Figure 2.**
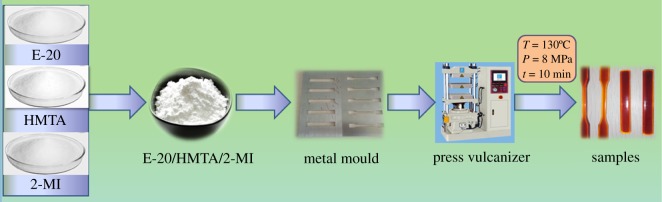
Flow chart showing the preparation of samples.

Powder epoxy resin (E-20), curing agent (HMTA) in a 100 : 8 equivalent ratio, together with 2-MI (0.6 wt% on the basis of the total weight of E-20 and HMTA) was stirred together until the homogeneous mixture was well blended (figure 1). Next, the mixed powder was poured into the preheated metal mould. Then, the metal mould was pressed by the press vulcanizer at 130°C for at least 10 min. Finally, the cured E-20/HMTA/2-MI systems were prepared and the average value was reported. All of the mechanical test samples were cured under the same conditions (figure 2).

### 2.3. Preparation of composites

Firstly, the reconstituted bamboo had to be dried under a preheated press vulcanizer at 100°C for 20 min. Secondly, appropriate amount of powder epoxy adhesive was uniformly coated on the reconstituted bamboo (BS) surface, fibreglass membrane (GF) surface and PET foam (PF) surface. Three kinds of materials were sequentially stacked based on the order of BS–GF–PF–GF–BS, and then pressed by press vulcanizer at 130°C for at least 10 min. Finally, the composites were obtained.

### 2.4. Characterization

#### 2.4.1. Fourier transform infrared analysis

FTIR spectra were recorded using a Perkin Elmer Model 1100 at room temperature. The wavelength range was from 4000 to 500 cm^−1^. Each powder sample was sandwiched between two KBr pellets. The spectra were obtained using 32 scans at a resolution of 4 cm^−1^.

#### 2.4.2. Thermogravimetric analysis

Thermogravimetric analysis (TGA) experiment was carried out on an STA 409PC with high purity as purge gas at a scanning rate of 20°C min^−1^ from room temperature to 800°C. Each sample of 5–10 mg in the initial weight was kept in an open Pt pan.

#### 2.4.3. Tensile test

The tensile properties of samples were characterized by an electronic universal testing machine with a 2 KN load cell at a cross-head speed of 2 mm min^−1^, according to the standard (GB/T1040.2-2006). The tensile strength, elongation at break and modulus of elasticity of both E-20/HMTA systems and E-20/HMTA/2-MI systems were obtained at room temperature. For each composition, the measurement was repeated with 10 samples.

#### 2.4.4. Impact test

Unnoticed impact strength tests were conducted on a pendulum impact testing machine according to the standard (GB/T1043-93). Each sample measuring 80 × 10 × 4 mm was used for this evaluation. For each composition, at least 10 samples were measured.

#### 2.4.5. Internal bond strength test

The internal bond strength of composites was characterized by an electronic universal testing machine, according to the standard (GB/T17657-2013). Each sample measuring 50 × 50 mm was used for this evaluation. For each composition, the measurement was repeated with 10 samples.

#### 2.4.6. Flexible strength test

The flexible strength of composites was performed by the electronic universal testing machine, according to the standard (GB/T1936.1-2009). Each sample measuring 300 × 25 × 25 mm was used for this evaluation. For each composition, the measurement was repeated with 10 samples.

#### 2.4.7. Dynamic mechanical analyser analysis

The dynamic mechanical properties at 1 Hz were measured using a TA Q800 dynamic mechanical analyser (DMA) in the single cantilever mode. Each sample measuring 35 × 10 × 3 mm was used for this evaluation. DMA measurements were performed at the heating rates of 3°C min^−1^ in a heat scan ranging from 60 to 210°C.

#### 2.4.8. Theory of curing kinetics

The differential scanning calorimetry (DSC) analysis was performed on a Netzsch STA 409PC Maia instrument under a nitrogen gas atmosphere. Dynamic DSC measurements were performed at the heating rates of 5, 10, 20 and 30 K min^−1^ in a heat scan ranging from 40 to 300°C to acquire curing heat-flow curves of the powder samples (in nitrogen flow of 50 ml min^−1^). The fresh samples of 5–10 mg in weight were kept in an aluminium pan under N_2_ atmosphere.

## 3. Results and discussions

### 3.1. Fourier transform infrared analysis

**Figure 3.**
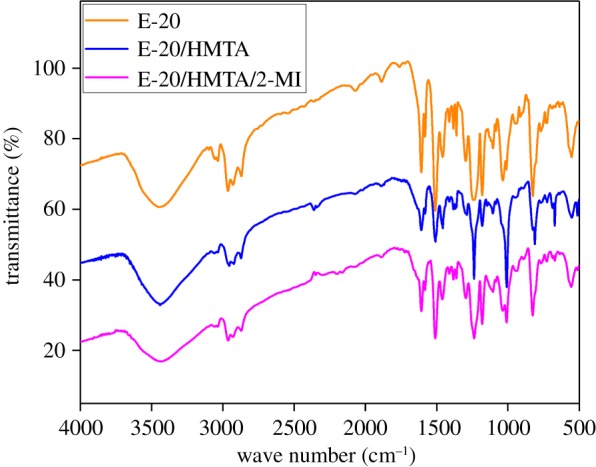
FTIR spectra of E-20, E-20/HMTA and E-20/HMTA/2-MI system.

FTIR spectra were often used to identify the changes in the chemical structure. Referring to the spectra of the E-20, E-20/HMTA and E-20/HMTA/2-MI systems shown in figure 3, differences in the spectra of the cured samples were mainly observed in the fingerprint region of the spectrum.

The peaks at 672 and 812 cm^−1^ were attributed to the bending vibration of C–N. The peak at 918 cm^−1^ was ascribed to the stretching vibration of epoxy structure [19]. The peaks at 1036 and 1181 cm^−1^ were associated with the ether bond stretching vibration. The peak at 2926 cm^−1^ was assigned to the methylene C–H stretching vibration. The peaks at 2872 and 2960 cm^−1^ were associated with the methyl C–H stretching vibration [20]. The peak at 3400 cm^−1^ was assigned to the –OH structure, which was attributed to the great hydroxyl bonding interaction of the phenol group [21].

**Figure 4.**
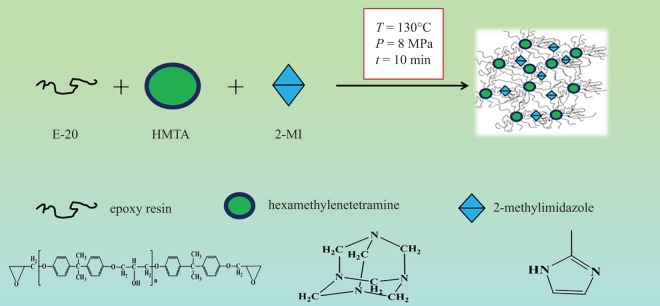
The model is the reticular cross-linking structure of the E-20/HMTA/2-MI system.

After modification, the peak of epoxy structure at 918 cm^−1^ gradually weakened and disappeared. The absorption peaks of –OH band at 3400 cm^−1^ weakened. These results indicated the successful ring-opening reaction of the epoxy group to form cross-linked polymers (figure 4).

The reaction equation of the E-20/HMTA systems was described as follows:


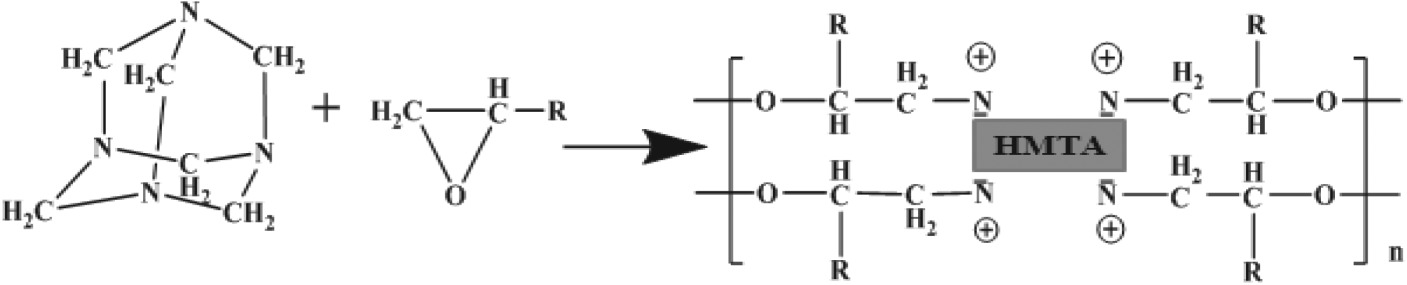


HMTA cured epoxy resin by anionic polymerization reaction. The anionic polymerization curing agent first acted on epoxy group, made it open ring, generated oxygen anion, and oxygen anion attacked epoxy group, which led to ring-opening addition. Finally, epoxy resins were cured by chain reaction [22].

The reaction equation of the E-20/2-MI systems was described as follows:


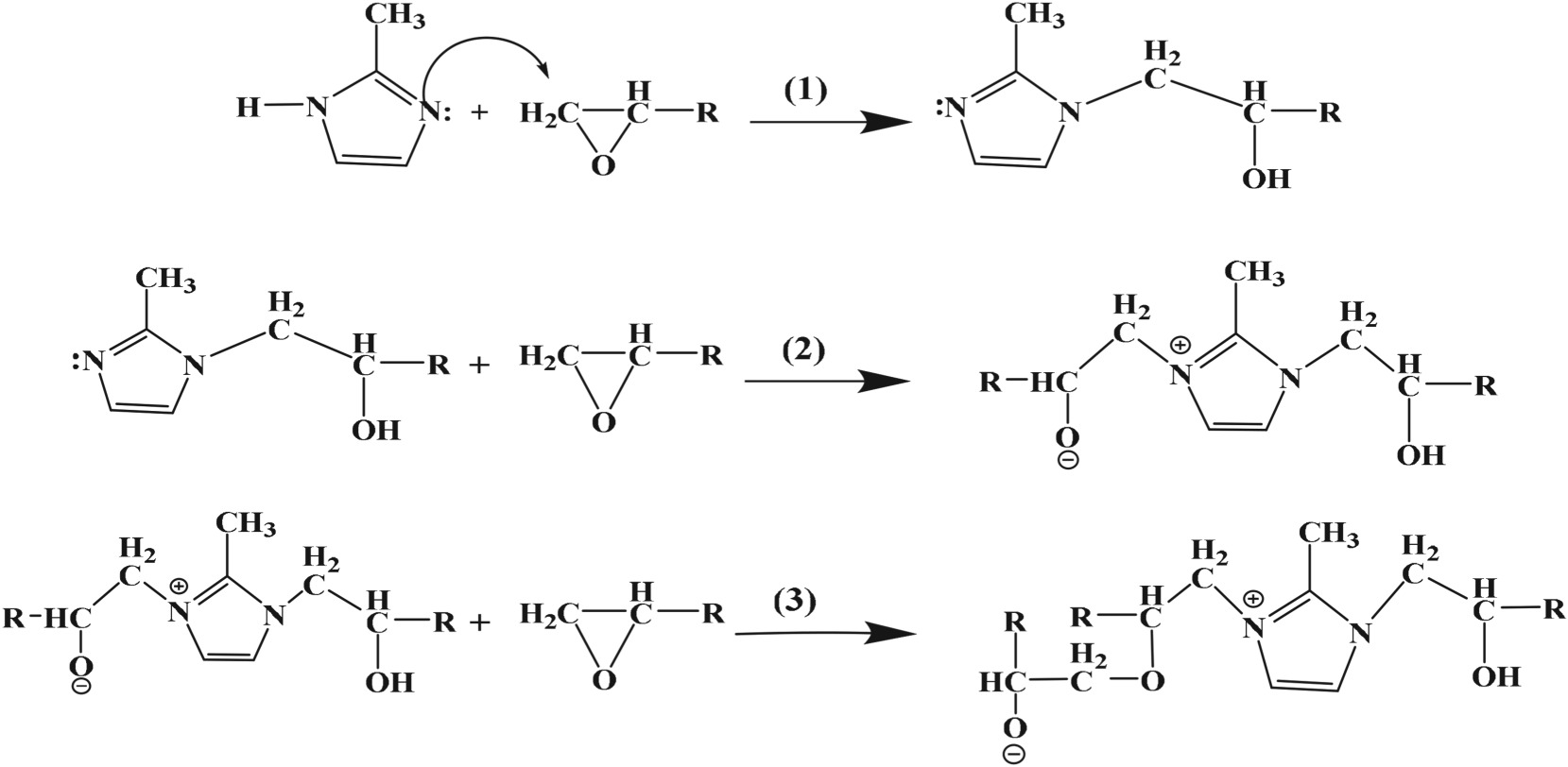


The curing reaction of the E-20/HMTA/2-MI systems took place in two steps. 2-MI as an anionic polymerization. Reaction mechanism: first, the active hydrogen of the secondary amine on imidazole was added to the epoxy group, and intramolecular complexes of positive and negative ions could be formed by the resulting adduct and another epoxy group. The negative ion at the centre of complex, which could catalyse the epoxies ring-opening reaction, finally reacted with curing agents and other epoxy groups [23].

### 3.2. Thermogravimetric analysis

**Figure 5.**
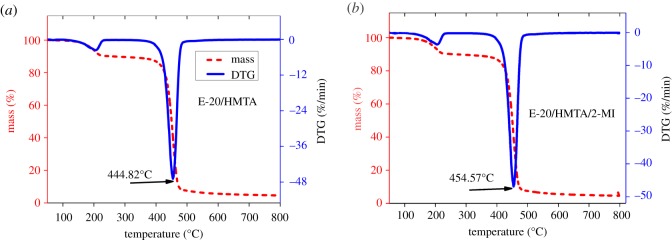
TGA stability of E-20/HMTA (*a*) and E-20/HMTA/2-MI (*b*) in N_2_ atmosphere.

TGA was a simple method to evaluate the degradation behaviours and thermal stability of polymers [24]. The degradation behaviours and thermal stability of cured E-20/HMTA/2-MI systems were compared with E-20/HMTA systems from thermogravimetric (TG) and derivative thermogravimetric analysis (DTG) in N_2_ atmosphere. The shapes of the derivative TGA curves (figure 5) for E-20/HMTA/2-MI systems were similar to those of E-20/HMTA, indicating that the structures in E-20/HMTA/2-MI systems were not significantly different from those of the E-20/HMTA systems in thermal stability [25]. There were two decomposition steps according to the two DTG peaks (figure 5*a*,*b*). Both the initial decomposition temperature (*T*_0_) and the maximum decomposition temperature (*T*_max_) of E-20/HMTA/2-MI increased. The E-20/HMTA system *T*_0_ was 173.32°C, lower than that of E-20/HMTA/2-MI system (185.17°C). The E-20/HMTA system *T*_max_ was 191.74°C and 444.82°C, respectively, lower than that of E-20/HMTA/2-MI system (205.32°C and 454.57°C). However, the E-20/HMTA system was about 7.66% char residues at 798.07°C, higher than that of E-20/HMTA/2-MI system (4.55%). The thermal stability of E-20/HMTA/2-MI system has been increased. The reason for the increase in the thermal stability of the E-20/HMTA/2-MI system was due to the incorporated 2-MI in the E-20/HMTA system. 2-MI could limit the intense motion of the polymer chain by different physico-chemical interactions.

### 3.3. Mechanical properties of the E-20/HMTA/2-MI systems

To investigate the toughening and reinforcing effects of the incorporated 2-MI in the E-20/HMTA systems, E-20/HMTA/2-MI systems with different 2-MI loadings were prepared and the mechanical properties were experimented, according to the m (E-20) : m (HMTA) : m (2-MI) = 100 : 8 : *X* ratio for the preparation of E-20/HMTA/2-MI systems (*X* = 0, 0.2, 0.4, 0.6, 0.8, 1).

**Figure 6.**
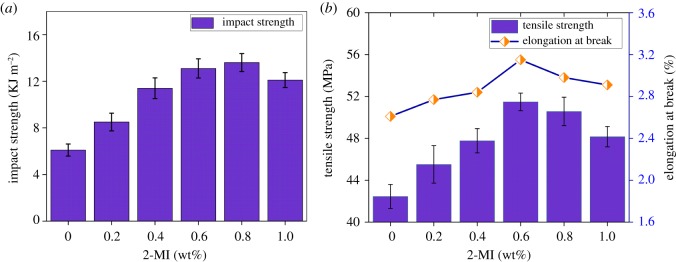
Effect of 2-methyl imidazole loadings on the mechanical properties of the E-20/HMTA/2-MI system. (*a*) Impact strength; (*b*) tensile strength and elongation at break.

As shown in figure 6*a*, it can be observed that the impact strength was obviously improved after adding 2-MI. The impact strength of E-20/HMTA/2-MI system up to 13.6 kJ m^−2^ at 0.8 wt% 2-MI loadings showed the best toughening effect, which was higher than that of E-20/HMTA system (6.1 kJ m^−2^). These data showed that the incorporation of 2-MI could effectively toughen the E-20/HMTA system.

The impact strength of the resulting E-20/HMTA/2-MI systems increased and then decreased as the ratios of E-20/HMTA/2-MI increased from 100/8/0 to 100/8/1. The further increase in 2-MI loadings over 0.8 wt% led to a decrease in the impact strength, this result was probably due to the cross-linking density reduction effect.

Tensile properties of tensile strength and elongation at break are shown in figure 6*b*. The average values of tensile and impact experiments-related parameters are shown in table 1. For most of the epoxy materials, improving toughness was achieved by sacrificing the stiffness [21]. However, compared with E-20/HMTA systems, the tensile experiment data of E-20/HMTA/2-MI systems showed that tensile strength and elongation at break were distinctly increased with the incorporation of 2-MI.

**Table 1.** Mechanical properties of impact strength, tensile strength and elongation at break.

2-MI (wt%)	impact strength (kJ m^−2^)	tensile strength (MPa)	elongation at break (%)
0	6.1 ± 0.52	42.45 ± 1.15	2.61 ± 0.12
0.2	8.5 ± 0.76	45.51 ± 1.79	2.77 ± 0.19
0.4	11.4 ± 0.89	47.77 ± 1.16	2.84 ± 0.13
0.6	13.1 ± 0.82	51.48 ± 0.84	3.15 ± 0.27
0.8	13.6 ± 0.77	50.57 ± 1.36	2.98 ± 0.21
1	12.1 ± 0.64	48.16 ± 0.97	2.91 ± 0.15

Tensile properties of the resulting E-20/HMTA/2-MI systems increased and then decreased as the ratios of E-20/HMTA/2-MI increased from 100/8/0 to 100/8/1. The tensile strength and elongation at break of E-20/HMTA/2-MI systems with 0.6 wt% 2-MI loadings were up to 51.48 MPa and 3.15%, respectively, which were 21.3% increase in tensile strength and 20.7% increase in elongation at break with respect to E-20/HMTA systems. Tensile strength was determined by the structure and cross-linking density [26]. The further increase in 2-MI loadings over 0.6 wt% led to a decrease in the tensile strength, this result was probably because the internal deformation ability of E-20/HMTA/2-MI system was enhanced, thus the cross-linking density was decreased.

Impact and tensile test indicated that incorporating 2-MI into the E-20/HMTA systems could improve toughness and stiffness. When the 2-MI loadings were under 0.6 wt%, elongation at break, impact strength and tensile strength all increase with 2-MI loadings. The increase in fractional free volume was beneficial to increase in impact strength and elongation at break, whereas the cross-linking density could be used to explain the increase in tensile strength [27]. Finally, the best mechanical properties were obtained at 100/8/0.6 ratio of E-20/HMTA/2-MI.

### 3.4. Dynamic mechanical analyser analysis

**Figure 7.**
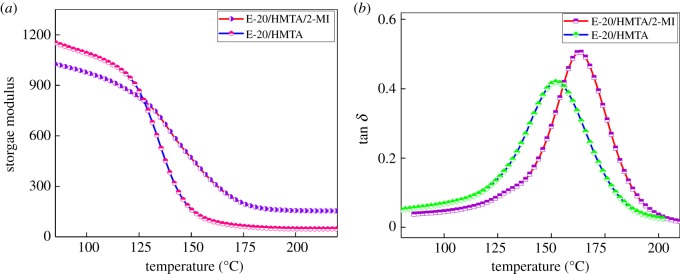
DMA analysis of E-20/HMTA (*a*) and E-20/HMTA/2-MI with 0.6 wt% 2-MI loadings (*b*).

The storage moduli (*E*′) and loss tangent (tan *δ*) as a function of temperature for the cured E-20/HMTA and E-20/HMTA/2-MI systems are shown in figure 7. For clarity, we only show data of E-20/HMTA systems and E-20/HMTA/2-MI systems with 0.6 wt% 2-MI loadings, which have biggest structure differences for easy comparison. E-20/HMTA and E-20/HMTA/2-MI systems showed a clear glass transition with the rubbery plateau modulus (Er). With the increasing temperature, the moduli reached the elastomeric region through the glass transition region, where more segments in the chains were moving in a cooperative manner [25]. According to the classical rubber elasticity, Er was proportional to the average cross-linking density [28].

The cross-linking density (*ρ*) of cured epoxy network can be calculated by the following equation:

3.1 $$\rho =\frac{\text{Er}}{3RT},$$

where *ρ*, Er, *R* and *T* are the cross-linking density per unit volume (mol cm^−3^), rubbery plateau modulus, the gas constant (8.314 J mol^−1^ K^−1^) and the absolute temperature, respectively. As shown in figure 7*a*, at 0.6 wt% 2-MI loadings, the cross-linking density of E-20/HMTA/2-MI systems increases by 16.5% compared with E-20/HMTA system (equation (3.1)). The increase in cross-linking density was due to the involvement of 2-MI in epoxy network as a chemical cross-linking point.

As shown in figure 7*b*, the curves of E-20/HMTA/2-MI systems were similar to that of E-20/HMTA systems and showed only one distinct peak, indicating that no sign of phase separation was found in the E-20/HMTA and E-20/HMTA/2-MI systems [29]. The half peak width of E-20/HMTA/2-MI systems was narrower than that of the E-20/HMTA systems, suggesting that cured E-20/HMTA/2-MI systems appear to be more uniform in structure than those of the E-20/HMTA systems [30]. Tan *δ* was defined as the ratio of loss modulus to the storage modulus, and the peak of the tan *δ* versus temperature curve was taken as *T*_g_. The E-20/HMTA/2-MI systems containing 0.6 wt% 2-MI showed *T*_g_ of 164.5°C, which was 12.5°C higher than the E-20/HMTA systems (152°C). *T*_g_ was affected by both the cross-linking density and the chain flexibility. As was mentioned before, the cross-linking density increased after adding 2-MI, the increase in *T_g_* was probably due to the structure of 2-MI, which enhanced the chain rigidity.

### 3.5. Cure kinetics of powder epoxy adhesive

**Figure 8.**
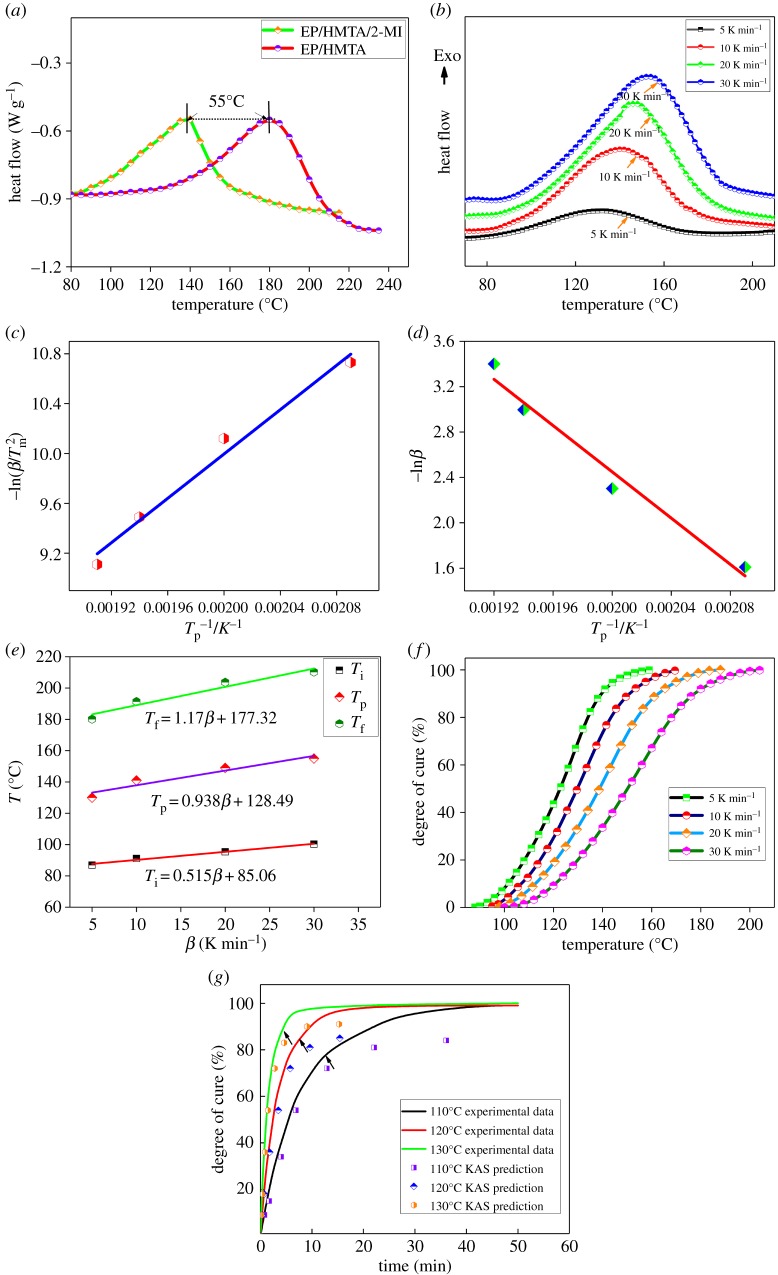
(*a*) DSC curves of E-20/HMTA and E-20/HMTA/2-MI system; (*b*) DSC profiles of E-20/HMTA/2-MI system for heat flow against temperature with heating rates of 5, 10, 20 and 30 K min^−1^; (*c*) linear plot of $-\text{ln }(\beta /T_{p}^{2})$ versus 1/*T*_p_ based on Kissinger's equation; (*d*) ln*β* versus 1/*T*_p_ based on Ozawa's theory; (*e*) *T*–*β* diagram; (*f*) degree of cure at different heating rates for the E-20/HMTA/2-MI systems; (*g*) model-free prediction of isothermal cure from 110 to 130°C using the KAS method.

Non-isothermal curing behaviours of E-20/HMTA systems with or without 2-MI were investigated by DSC. With the introduction of 2-MI into the E-20/HMTA system, the exothermic peak of E-20/HMTA system was about 55°C higher than that of E-20/HMTA/2-MI system as shown in figure 8*a*, which indicated that the incorporation of 2-MI could decrease the activation energy and increase the rate of curing reaction.

A curing reaction kinetics study of E-20/HMTA/2-MI systems played an important role in optimizing curing reaction cycles [31]. Figure 8*b* exhibits DSC curves of E-20/HMTA/2-MI systems at different heating rates (5, 10, 20 and 30 K min^−1^). Each curve showed a single exothermic peak corresponding to the ring-opening reaction between epoxy and amine. The exothermic curing reaction of E-20/HMTA/2-MI systems occurred in the temperature range of 85–177°C. As shown in table 2, the peak curing temperature shifted with different heating rates. With an increase in heating rate, the initial and peak temperature were higher, the curing time could be reduced greatly and have a broader range of curing temperature, because the thermal effect per unit time was increased, it could have a greater thermal inertia, and the onset and peak temperature deviated to high temperature direction with an increase in heating rate [9].

**Table 2.** The exothermic peak temperature of the E-20/HMTA/2-MI system at different heating rates. (*T*_i_, initial temperature; *T*_p_, peak temperature; *T*_f_, final temperature).

*β* (K min^−1^)	*T*_i_ (K)	*T*_p_ (K)	*T*_f_ (K)
5	360.05	403.15	453.25
10	364.25	414.15	464.55
20	368.55	422.15	476.85
30	373.45	428.15	483.35

The activation energy of the cure reaction was determined by both Kissinger's [32] and Ozawa's [33] methods at any heating rates. The Kissinger equation was the most extensively used method to calculate the activation energy. Kissinger's equation can be expressed as follows:

3.2 $$\ln\;\left(\frac{\beta }{T_{p}^{2}}\right)=\ln\;\left(\frac{RA}{E}\right)-\frac{E_{a}}{RT_{p}}$$

and

3.3 $$\;A=\frac{\beta \cdot E_{a}\exp(E/T_{P})}{RT_{P}^{2}},$$

where *R*, *E*_a_ *β* and *T*_p_ are the gas constant (8.314 J mol^−1^ K^−1^), apparent activation energy, heating rate and exothermic peak temperature, respectively. Among them, *E*_a_ can be obtained from the plot and the intercept of −ln$\left(\beta /T_{p}^{2}\right)$ versus 1/*T*_p_.

Ozawa's equation can be expressed as follows:

3.4 $$\frac{\text{d}(\ln\beta )}{\text{d}(1/T_{p})}=-\frac{1.052E_{a}}{R}.$$

Crane's equation can be expressed as follows:

3.5 $$\frac{\text{d}(\ln\beta )}{\text{d}(1/T_{p})}=-\frac{E_{a}}{nR}-2T_{p},$$

where *R*, *E*_a_, *β*, *T*_p_ and *n* are the gas constant (8.314 J mol^−1^ K^−1^), apparent activation energy, heating rate, exothermic peak temperature and curing reaction order, respectively. Among them, *n* can be obtained from the plot of ln*β* versus 1/*T*_p_.

The plot of $-\text{ln}(\beta /T_{p}^{2})$ versus 1/*T*_p_ based on Kissinger's equation, as well as ln*β* versus 1/*T*_p_ based on Ozawa's theory are shown in figure 8*c*,*d*. According to Kissinger's method: the activation energy (*E*_a_) and pre-exponential factor (*A*) could be obtained from the plot and the intercept of $-\text{ln}(\beta /T_{p}^{2})$ versus 1/*T*_p_, the activation energy and pre-exponential factor were about 100.3 kJ mol^−1^ and 3.57 × 10^11^ s^−1^, respectively (equations (3.2) and (3.3)). Ozawa's method: the activation energy could be calculated by the plot of ln*β* versus 1/*T*_p,_ the activation energy was 107.25 kJ mol^−1^ (equation (3.4)). It can be seen that the apparent activation energy acquired using Ozawa's equation was higher than that of Kissinger's equation [34–36]. E-20/HMTA/2-MI systems have a lower activation energy and higher catalytic activity, because epoxy groups were attached to oxygen atoms, the inductive effect could increase the polarization degree of epoxy groups, the ring-opening reaction of epoxy groups was promoted. According to the Crane method, the curing reaction order of the E-20/HMTA/2-MI system was 0.94 (equation (3.5)).

As shown in figure 8*e*, the epitaxial method was used to obtain *T*_i_, *T*_p_ and *T*_f_ when *β* = 0 (onset temperature (*T*_i_), peak temperature (*T*_p_) and post-treatment temperature (*T*_f_)). *T*_i_, *T*_p_ and *T*_f_ temperatures could be used as the actual curing reference temperature, combined with the actual situation to determine the curing conditions [37]. According to the extrapolation curve of the E-20/HMTA/2-MI system, it could be concluded that *T*_i_ = 0.515*β* + 85.06, *T*_p_ = 0.938*β* + 128.49, *T*_f_ = 1.17*β* + 177.32. By extrapolating the *β* to 0, the *T*_i_, *T*_p_ and *T*_f_ were obtained at 85.06°C, 128.49°C and 177.32°C, respectively. Optimal curing parameter was suggested to be from 130°C.

For that, various curing degrees (*α*) were evaluated by measuring the total area (d*H*_T_) under the DSC curve and partial area (d*H*) at a definite temperature. The degree of curing (*α*) is the ratio of d*H* to d*H*_T_ [38]. A transformation was performed to convert non-isothermal DSC scans into degree of cure versus isoconversional temperatures at different heating rates. A representative of this transformation is exhibited in figure 8*f* for the E-20/ HMTA/2-MI system. The curing rate was slow due to the induction period of reaction at a lower temperature. Then, the curing rate reaches maximum value. Finally, the curing rate decreases gradually. Curing degree and temperature showed ‘S’ curve during the whole curing process.

The Vyazovkin method uses the following equations:

3.6 $$g(\alpha )=t_{\alpha }A_{\alpha }\exp\left(\frac{-E_{a}}{RT_{0}}\right)$$

and

3.7 $$\;g(\alpha )=\left(\frac{A_{\alpha }}{\beta }\right)\int_{T_{0}}^{T_{\alpha }}\exp\left(\frac{-E_{a}}{RT_{0}}\right)\text{d}T.$$

Equations (3.6) and (3.7) stand for the isothermal (*T*_0_ = const) and non-isothermal (*β* = const) conditions, respectively. To obtain *t_α_*, equation (3.7) can be divided by equation (3.6).

3.8 $$t_{\alpha }=\frac{\left[\int_{T_{0}}^{T_{\alpha }}\exp(-E_{a}/RT)\text{d}T\right]}{\beta \exp(-E_{a}/RT_{0})}.$$

Equation (3.8) was a unified model-free method, which can enable the determination of time at which a given conversion will be reached at any temperature and will be computed [39]. Figure 8*g* shows the isothermal curing reaction of E-20/HMTA/2-MI systems cured from 110 to 130°C as predicted by using the conversion-dependent apparent activation energy curve (*E*_a_). When the conversion rates were greater than 50%, the curing time of KAS prediction was lower than the actual reaction time.

### 3.6. Applications of powder epoxy adhesives in bamboo wood-based panel

**Figure 9.**
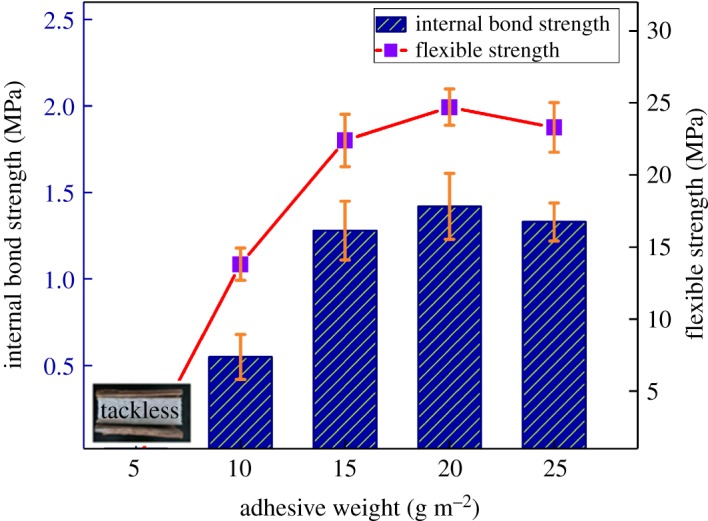
Effect of powder epoxy adhesives loadings on the internal bond strength and flexible strength of the bamboo wood-based panel composites.

Effects of epoxy adhesive amount on the mechanical properties of bamboo wood-based panel were evaluated. Internal bond strength and flexible strength of bamboo wood-based panel composites with different powder epoxy adhesives loadings are shown in figure 9. It can be observed that the internal bond strength and flexible strength were obviously improved after adding powder epoxy adhesives. The bamboo wood-based panel composites with 20 g m^−2^ powder epoxy adhesive loadings show the best internal bond strength and flexible strength. At 5 g m^−2^ powder epoxy adhesive loadings, the composites will generate debonding and bursting. Internal bond strength and flexible strength were both decreased when the powder epoxy adhesive loadings were over 20 g m^−2^, indicating that the amount of powder epoxy adhesives was increased too much, which caused thickening of the adhesive layer and increasing of the stress [40]. Finally, in consideration of the cost, the best powder epoxy adhesive loadings were obtained at 15 g m^−2^.

## 4. Conclusion

In this work, we have successfully synthesized a series of fast-curing powder epoxy adhesives. The structure and properties of the E-20/HMTA/2-MI systems were characterized by FTIR, TGA, DMA and DSC. With the development of curing reaction, the peak of epoxy structure at 918 cm^−1^ gradually weakened and disappeared and the absorption peaks of –OH band at 3400 cm^−1^ weakened. These results indicated the successful ring-opening reaction of the epoxy group to form cross-linked polymers. Impact and tensile test indicated that the incorporated 2-MI in the E-20/HMTA systems could improve toughness and stiffness. The main reason was that 2-MI as a Lewis base, led to a higher cross-linking density by anionic chain-growth reaction mechanism. The best mechanical properties were obtained at 100/8/0.6 weight ratio of E-20/HMTA/2-MI systems. The TGA results showed that E-20/HMTA/2-MI systems have a higher thermal stability in comparison with the E-20/HMTA systems. The main reason was that the incorporated 2-MI in the E-20/HMTA systems could limit the intense motion of the polymer chain by different physico-chemical interactions. DSC experiments revealed that with the introduction of 2-MI into the E-20/HMTA system, the exothermic peak of E-20/HMTA/2-MI system was about 55°C lower than that of E-20/HMTA system. The main reason was that the incorporated 2-MI in the E-20/HMTA systems could decrease the activation energy and increase the rate of curing reaction. Based on Kissinger and Crane kinetic analysis, the action energy and the order of curing reaction were obtained by kinetic calculation, 100.3 kJ mol^−1^ and 0.94, respectively. According to the KAS method, the curing time of the E-20/HMTA/2-MI systems was predicted by evaluating a relationship between temperature and curing time. Because of these attractive properties as well as advantages of non-toxicity, ease of use and high recycling rate, powder epoxy adhesive was valuable for industrial application.

## Data accessibility

All the data are provided in the main text.
